# Non‐Cyclical Mastalgia as a Central Sensitization Component: Implications for Multidisciplinary Treatment Approaches

**DOI:** 10.1002/brb3.70962

**Published:** 2025-10-11

**Authors:** Pelin Basım, Sena Tolu, Caglar Kazim Pekuz, Perya Abbasoglu, Serdar Basım

**Affiliations:** ^1^ Department of General Surgery Medipol University Istanbul Turkey; ^2^ Department of Physical Medicine and Rehabilitation Medipol University Istanbul Turkey; ^3^ Esenyurt Necmi Kadıoglu State Hospital Department of General Surgery Istanbul Turkey; ^4^ Department of General Surgery, Basaksehir State Hospital Istanbul Turkey

**Keywords:** breast pain, central sensitization, chronic pain, innovative treatments, non‐cyclical mastalgia, psychosocial factors, quality of life

## Abstract

**Objectives:**

Non‐Cyclical mastalgia (NCM) is a chronic breast pain condition in women of reproductive age, often linked to anxiety and reduced quality of life (QoL). Evidence suggests central sensitization (CS) may contribute to NCM, but its clinical significance and management remain unclear.

**Methods:**

This cross‐sectional study included 201 women aged 25 to 65, with 106 NCM patients and 95 healthy controls, to investigate the association between NCM and central sensitization syndromes (CSS). Data collection involved demographic characteristics and assessments using the Central Sensitization Inventory (CSI), Nottingham Health Profile (NHP), Short‐Form McGill Pain Questionnaire (SF‐MPQ), Pain Detect, Leeds Assessment of Neuropathic Symptoms and Signs (LANSS), and Hospital Anxiety and Depression Scale (HADS). The main outcome is to reveal the interrelationship between NCM and CSS.

**Results:**

NCM patients had significantly higher Central Sensitization Inventory‐A (CSI‐A) scores than controls (*p* < 0.001), with 15.1% surpassing the critical threshold of 40, indicating pronounced CS. NCM patients also had increased NHP‐1, NHP‐2, and HADS‐total scores (*p* = 0.008, *p* = 0.003, *p* = 0.004), reflecting greater distress. Subgroup analyses showed more intense pain (*p* < 0.001) and sleep disturbances (*p* = 0.002). CSI‐A scores strongly correlated with SF‐MPQ, LANSS, Pain Detect, and HADS (all *p* < 0.001). Regression analysis identified pain duration, Pain Detect, and SF‐MPQ sensory scores as key predictors of CSI‐A (*p* < 0.001).

**Discussion:**

NCM shares characteristics with chronic pain syndromes linked to CSS. Multidisciplinary approaches and innovative treatments, such as cognitive therapies combined with integrative approaches may improve outcomes.

## Graphical abstract

This cross‐sectional study included 201 women, comprising 106 patients with non‐cyclical mastalgia (NCM) and 95 healthy controls, to analyze the correlation between NCM and central sensitization syndromes (CSS). NCM patients showed higher central sensitization scores and a greater burden of pain, anxiety, depression, and sleep disturbances compared to controls. Central sensitization was closely linked with pain severity and psychological distress, with pain duration and sensory symptoms emerging as key predictors. NCM shares characteristics with chronic pain syndromes linked to CSS. These findings highlight the need for multidisciplinary management strategies, including cognitive and integrative therapies, to improve outcomes in NCM.

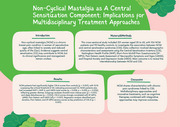

## 1 Introduction

Mastalgia is the most common breast‐related symptom among women, with approximately 15% to 50% of visits to breast clinics attributed to this complaint. (Smith et al. 2004) Although cyclical mastalgia (CM), the most common form of “breast pain,” typically occurs during premenstrual periods, non‐cyclical mastalgia (NCM) is also a significant concern among women of reproductive age, affecting approximately 10% to 25% of women at least once or more during their lifetime. (Maddox et al. 1989; Hacimusalar et al. 2020; Bolat et al. 2022) Despite the use of advanced imaging methods to rule out breast cancer, NCM‐related discomfort still causes patients to experience fear and anxiety about breast cancer, leading to an increased frequency of visits to breast clinics. Unlike CM, NCM is less clearly associated with the menstrual cycle, often localized to one breast or a specific area, continuous in nature, and resistant to treatments effective for CM. These features disrupt daily activities and contribute to psychosocial challenges for affected individuals. (Hacimusalar et al. 2020; Bolat et al. 2022; Cornell et al. 2020) Existing literature emphasizes that a chronic inflammatory process plays a major role in the development of NCM, rather than a causal hormonal hypothesis. (Bolat et al. 2022; Cornell et al. 2020; Jokich et al. 2017) Potential risk factors include a history of previous pregnancy, lactation, intramammary thrombophlebitis, fibrocystic breast changes, benign tumors, duct ectasia, side effects of some medications, stretching of Cooper's ligaments, a diet with poor nutritional quality, higher‐risk lifestyle factors, and uncontrolled hormone replacement therapy. However, these risk factors are present in only a small proportion of individuals with the condition. (Bolat et al. 2022). (Jokich et al. 2017; Groen et al. 2017) Over 90% of NCM cases lack identifiable breast pathology, similar to other chronic pain disorders without clear anatomical causes. This unresolved etiology has shifted researchers' focus to psychosocial factors that may contribute to NCM. Studies have reported a significant correlation between NCM and anxiety, depression, and sleep disorders, suggesting that sensitization processes may play a role in its pathogenesis (Groen et al. 2017; Ader et al. 2001; Yılmaz et al. 2014; Basım and Tolu 2022).

“Sensitization” refers to changes in nociceptive neurons present in several different types of chronic pain syndromes, especially in neuropathic conditions. Peripheral sensitization (PS) occurs when nociceptors become hyper‐responsive due to tissue injury or inflammation, increasing pain perception. The sensitized nociceptors then transmit signals to the spinal cord and higher brain centers, leading to pain perception (Fernández‐de‐Las‐Peñas et al. 2020; Nijs et al. 2021).

Central sensitization syndrome (CSS) refers to an enhanced responsiveness of nociceptive neurons in the central nervous system (CNS) to their usual or subthreshold afferent stimuli. Due to the hyperexcitability of CNS neurons, pain can be triggered even with minimal or no tissue damage. This can result in heightened pain sensitivity, amplification of pain signals, and the development of persistent pain states. CSS is believed to be driven by prolonged nociceptive input from the periphery, leading to changes in the structure and function of neurons in the spinal cord and brain. The relationship between PS and CSS is bidirectional. PS can lead to the development of CSS, as prolonged nociceptive input from the periphery drives changes in central neurons. Conversely, central sensitization can enhance PS by increasing the release of inflammatory mediators (Fernández‐de‐Las‐Peñas et al. 2020; Nijs et al. 2021; Finnerup et al. 2022; Treede et al. 2022). This bidirectional and mutual interaction may explain the pain characteristics observed in NCM. Despite the resolution of all potential underlying etiological factors, the persistence of pain without significant reduction in a subset of patients suggests that NCM may chronically evolve through mechanisms distinct from causally driven pain, resembling other sensitized pain conditions. Thus, regardless of the underlying etiological factor, it may be hypothesized that, similar to other subgroups of CSS, NCM could transform into a chronic pain syndrome in a specific group of patients. This study aims to identify whether NCM may be classified as a component of CSS and to assess its prevalence alongside other CSS conditions.

## 2 Materials and Methods

### 2.1 Study Design and Patients

This cross‐sectional single‐centered study includes female patients aged 25 to 65 years who presented to the Medipol University Medical Faculty general surgery outpatient clinic between December 2020 and April 2023. In order to establish the study groups, a total of 723 patients with similar demographic characteristics who presented to the General Surgery Clinic of Medipol University Faculty of Medicine Hospital—either with breast pain and a diagnosis of non‐cyclic mastalgia (NCM) for at least 6 months or with other general surgical complaints—were included in the study. All patients were screened with physical breast examinations and compatible radiological, noninvasive imaging modalities matched with patients’ ages. In the study, 201 individuals met the inclusion criteria; 106 with NCM and 95 healthy controls were included in the study. The sample size was determined pragmatically, with all patients agreeing to participate over the study period.

All participants underwent a comprehensive physical breast examination by the same breast surgeon to identify any lumps, nipple retraction or discharge, skin changes, or axillary lymphadenopathy. Radiological breast imaging, including ultrasound, mammography, and magnetic resonance imaging (MRI), was also conducted. The physical examination, breast imaging, and medical history questionnaire were essential components of the comprehensive assessment performed on all participants to ensure accurate diagnosis of NCM. All the participants of the study were also evaluated by the same physical therapist of the university hospital to evaluate any associated musculoskeletal system disorder radiating to the breast or chest wall area. This multidisciplinary approach, involving a breast surgeon and a physical therapist, aimed to provide a thorough evaluation of participants' breast pain and minimize potential biases that could affect study outcomes.

The exclusion criteria were as follows: (i) Age < 18 or > 65 years, (ii) pregnancy or recent lactation, (iii) previous breast biopsy or surgical procedures for benign breast disease or breast cancer, (iv) refusal to participate, (v) any breast pathology diagnosed during the study period, including cysts, solid lesions, or infections, and (vi) a history of cognitive dysfunction or psychiatric disorders, as well as current psychiatric treatment that could impair the ability to complete questionnaires accurately.

### 2.2 Ethical Considerations

The study was performed in accordance with the Declaration of Helsinki and approved by the ethical committee of the Istanbul Medipol University on December 12, 2020, with the reference number E‐10840098‐772.02‐66560. All subjects were required to provide written informed consent before participating.

### 2.3 Data Collection and Questionnaires

Demographic information, including age, body mass index (BMI), employment status, education level, monthly income, smoking status, medical comorbidities, chronic drug use, and duration of pain, was obtained from all participants. For the patients with NCM, pain characteristics were assessed using a specifically designed breast pain questionnaire, the Short‐Form McGill Pain Questionnaire (SF‐MPQ). Originally developed by Melzack in 1975 and revised in 1987 to enhance its practicality in clinical settings, the SF‐MPQ is a widely used questionnaire in both clinical and research settings to evaluate the underlying mechanism of breast pain. The SF‐MPQ comprises three key components: the Visual Analog Scale (VAS) of Pain Intensity, the Present Pain Intensity (PPI) Score, and Verbal Descriptors, which include sensory and affective dimensions. This multidimensional approach evaluates both the overall and present pain intensity while distinguishing between nociceptive and sensory aspects and exploring the emotional components of pain (Melzack 1987).

Mastalgia, being a subjective symptom, poses a challenge for accurate measurement due to its inherently personal and subjective nature. To address this challenge and enhance the objectivity of pain assessment, we intended to incorporate the Pain Detect and Leeds Assessment of Neuropathic Symptoms and Signs (LANSS) pain scales alongside SF‐MPQ. Another important point that we considered about these scales is that they are specifically designed to evaluate neuropathic pain, providing a structured approach to quantify pain severity and characteristics.

The Pain Detect questionnaire helps to differentiate between nociceptive and neuropathic pain components by assessing the intensity and nature of the pain experienced. It includes questions about the location, frequency, and quality of pain, as well as factors that exacerbate or relieve it. The LANSS pain scale, on the other hand, combines both a questionnaire and a physical examination component to evaluate pain based on sensory abnormalities and the presence of dysesthesia, allodynia, or other neuropathic pain indicators. By integrating these scales, the study aims to provide a more comprehensive and nuanced assessment of NCM, acknowledging its subjective nature while striving for greater objectivity. This multi‐faceted approach allows for better differentiation between pain types and can contribute to more targeted and effective treatment strategies.

The Central Sensitization Inventory (CSI) is a self‐reported questionnaire designed to assess symptoms associated with central sensitization (CS). It was developed and published by Mayer et al. (2012). The CSI consists of two parts: Part A includes 25 items evaluating common symptoms like chronic pain, fatigue, and sleep disturbances, while Part B identifies related medical diagnoses such as fibromyalgia or irritable bowel syndrome. The tool helps clinicians understand the impact of central sensitization on patients and guide tailored treatment approaches.

The Nottingham Health Profile (NHP) is a well‐established tool for evaluating the quality of life (QoL) in individuals, particularly in the context of chronic conditions. The NHP consists of two parts, each focusing on different aspects of an individual's life affected by health. NHP‐1 evaluates domains, including sleep, physical mobility, energy, pain, emotional reactions, and social isolation. Participants score items within each section, and the scores are rescaled to a range of 0 to 100. This allows for a quantitative assessment of the impact of health on different aspects of daily living. NHP‐2 focuses on specific areas of life that are most affected by health, including employment, household activities, social life, home life, sex life, hobbies and interests, and holidays. Participants indicate whether their present health status has affected each of these areas. The Turkish version of the NHP, validated in 2000, was used in this study; it maintains the structure and purpose of the original tool. The decision to use the Turkish version aligns with the goal of assessing the QoL by perceived health status in individuals with chronic diseases. The reported high internal consistency (Cronbach's *α* = 0.87) and reliability (0.88) for the Turkish version, similar to the original version, indicate that the translated tool is reliable and internally consistent. This suggests that the Turkish version of NHP provides consistent and dependable results when measuring the impact of health on different aspects of QoL (Kucukdeveci et al. 2000).

The use of the Hospital Anxiety and Depression Scale (HADS) in assessing anxiety and depression levels is a common and effective practice in medical settings. The study employed the Turkish version of HADS, validated for the Turkish population. HADS is described as a simply administered form, which is beneficial in clinical and research settings where ease of use is crucial. The 14‐item Likert‐type checklist allows patients to respond based on a 4‐point scale (ranging from 0 to 3) to indicate the presence and severity of symptoms they experienced in the past week. HADS is divided into two subscales, each consisting of seven questions. One subscale assesses anxiety symptoms, and the other assesses depression symptoms; in this context the use of the Turkish version of HADS provides a standardized, validated psychometric method for assessing anxiety and depression levels in NCM patients (Zigmond and Snaith 1983).

### 2.4 Statistical Analysis

Data were analyzed using IBM SPSS for Windows, version 23.0 (IBM Corp., Armonk, NY, USA). The Shapiro–Wilk test was used to assess the normality of the data distribution. Descriptive statistics, including frequency, percentage, mean, median, standard deviation, minimum, and maximum values, were calculated to summarize the characteristics of the data.

In paired group comparisons, categorical variables were compared using the chi‐square test, normally distributed continuous data were compared using the independent‐samples *t*‐tests, and non‐normally distributed continuous data were compared using the Mann–Whitney *U* test, which is a non‐parametric test for comparing two independent groups.

Correlations between variables were evaluated using either Pearson's correlation coefficient (for normally distributed data) or Spearman's rank correlation coefficient (for non‐normally distributed data). A significance level of *p* < 0.05 was applied, indicating that results with a probability of occurring by chance less than 5% were considered statistically significant.

## 3 Results

A total of 201 women were enrolled in the study, including 106 patients suffering from NCM and 95 individuals without any breast complaint admitted to the breast surgery clinic for regular breast examination within the specified time interval. The two groups were matched for demographic characteristics.

Table 1 compares the demographic characteristics and central sensitization scores (CSI‐A) of patients with and without NCM (Group 1—control group) (Group 2–NCM patients). CSI‐A scores of Group 2 patients were statistically significantly higher than those of Group 1, namely the control group (*p* < 0.001). Group 2 has been found to consist of statistically significantly lower BMI patients, and the percentage of working class was found to be significantly higher compared to Group 1 (*p* = 0.011, *p* < 0.001, respectively). The group of employees working in the private sector has inarguably been determined as the subgroup that affects this statistical difference the most (p < 0.003).

**TABLE 1:** Comparison of demographic features and CSI‐A of patients with and without NCM (Group 1‐ control group/without NCM) (Group 2‐NCM patients).

		Group 1 (*n* = 95)	Group 2 (*n* = 106)	p
**Age** (mean ± S.D.)		32.54 ± 5.16	31.67 ± 5.84	0.289
**BMI** (mean ± S.D.)		26.36 ± 3.78	24.82 ± 1.89	**0.011**
**Occupation** *n (%)*	**Homemaker/not employed outside home**	4 (4.2)	31 (29.2)	**< 0.001**
	**Public sector employee**	20 (21.1)	57 (53,8)	
	**Private sector employee**	49 (51.6)	14 (13.2)	
	**Retired**	22 (23.2)	4 (3.8)	
**Education** *n (%)*	**No formal education**	9 (9.5)	12 (11.3)	0.972
	**Primary/secondary school**	17 (17.9)	19 (17.9)	
	**High school**	43 (45.3)	48 (45.3)	
	**College/University**	26 (27.4)	27 (25.5)	
**Income status** ^a^ *n (%)*	**Low income** **(< 20,000 TRY)**	13 (13.7)	20 (18.9)	0.554
	**Middle income** **(20,000–50,000 TRY)**	57 (60.0)	57 (53.8)	
	**High income (> 50,000 TRY)**	25 (26.3)	29 (27.4)	
**Smoking status** *n (%)*	**No**	22 (23.2)	29 (27.4)	0.783
	**Yes**	38 (40.0)	41 (38.7)	
	**Quit**	35 (36.8)	36 (34.0)	
**Medication use** *n (%)*	**No**	88 (92.6)	94 (89.5)	0.443
	**Yes**	7 (7.4)	11 (10.5)	
**Chronic illness** *n (%)*	**No**	87 (91.6)	88 (83.0)	0.071
	**Yes**	8 (8.4)	18 (17.0)	
**CSI—A** (mean ± S.D.)		12.12 ± 5.18	24.25 ± 13.46	**< 0.001**

^a^ The official currency of the Republic of Turkey is the Turkish Lira (TRY). During the period in which the study was conducted, in the Republic of Turkey, the minimum wage was 22.104 (TRY) (approximately 550 USD).

A comparison of clinical parameters about physical and mental well‐being for NCM patients and the control group is given in Table 2. When Groups 1 and 2 were compared, there was a statistically significant difference in terms of NHP‐1, NHP‐2, and HADS‐total scorings of the patients (*p* = 0.008, *p* = 0.003, and *p* = 0.004, respectively). All three parameters were found to be significantly higher in NCM patients. Another important interference of the results showed that among all the subgroups of NHP‐1, the pain and sleep scores were found to be significantly higher in the NCM group compared to the control group (*p* < 0.001 and *p* = 0.002).

**TABLE 2:** Comparison of clinical parameters about physical and mental well‐being for NCM patients and control group.

	Group 1 (*n* = 95)		Group 2 (*n* = 106)		p
	Mean ± SD	Median (Min–Max)	Mean ± SD	Median (Min–Max)	
**Nottingham Health Profile Part 1, median (min–max)**	69.56 ± 55.08	57.89 (0.00–295.38)	108.94 ± 97.64	75.06 (9.76–443.44)	**0.008**
Pain	6.87 ± 9.80	0.00 (0.00–33.77)	30.30 ± 20.90	24.13 (0.00–83.68)	**< 0.001**
Emotional reactions	16.80 ± 14.04	10.47 (0.00–54.37)	17.37 ± 16.02	13.95 (0.00–64.38)	0.947
Sleep	8.85 ± 14.48	0.00 (0.00–77.63)	20.50 ± 26.69	12.57 (0.00–100.00)	**0.002**
Social isolation	11.41 ± 20.22	0.00 (0.00–77.63)	11.78 ± 23.72	0.00 (0.00–100.00)	0.439
Physical mobility	9.08 ± 22.97	0.00 (0.00–67.16)	7.58 ± 14.43	0.00 (0.00–67.16)	0.089
Energy	16.63 ± 22.97	0.00 (0.00–100.00)	20.49 ± 31.82	0.00 (0.00–100.00)	0.939
**Nottingham Health Profile Part 2, median (min–max)**	1.09 ± 1.02	1.00 (0.00–5.00)	1.75 ± 1.55	2.00 (0.00–6.00)	**0.003**
**HADs—total, median (min–max)**	13.13 ± 4.34	13.00 (6.00–24.00)	15.63 ± 5.75	14.00 (8.00–33.00)	**0.004**
HADs‐anxiety	6.54 ± 2.36	6.00 (3.00–13.00)	7.79 ± 3.97	7.00 (1.00–19.00)	0.052
HADs‐depression	6.57 ± 2.70	7.00 (2.00–15.00)	7.94 ± 2.76	8.00 (3.00–17.00)	**0.001**

Table 3 presents a comparison of CSI parameters (both parts A and B) for NCM patients and the control group. In Group 1, none of the patients has a CSI‐A score exceeding 40, which is a commonly used—but without clear boundaries—threshold in CSS diagnosis. On the other hand, 15.1% (*n* = 16) of the Group 2 patients described a CSI‐A score exceeding 40. The statistical analysis showed a clearly significant difference in favor of Group 2, presenting a significantly higher CSI‐A score (p < 0.001). In support of this finding, when comparing the disease subgroups that make up CSI‐B, chronic fatigue syndrome, fibromyalgia, temporomandibular joint syndrome, migraine or tension headache, irritable bowel syndrome, and anxiety and depression scores were statistically significantly higher and associated closely with NCM (*p* < 0.001, *p* = 0.010, *p* = 0.017, *p* = 0.005, *p* = 0.001, *p* = 0.011, and *p* < 0.001, respectively).

**TABLE 3:** Comparison of CSI parameters (part 1 and 2) for NCM patients and control group.

		Group 1 (*n* = 95)	Group 2 (*n* = 106)	*p*
**CSI—A** *n (%)*	**CS Score < 40**	95 (100.0)	90 (84.9)	**< 0.001**
	**CS Score ≥ 40**	0 (0.0)	16 (15.1)	
**Restless leg syndrome** *n (%)*	**No**	89 (93.7)	92 (86.8)	0.103
	**Yes**	6 (6.3)	14 (13.2)	
**Chronic fatigue syndrome** *n (%)*	**No**	81 (85.3)	59 (55.7)	**< 0.001**
	**Yes**	14 (14.7)	47 (44.3)	
**Fibromyalgia** *n (%)*	**No**	85 (89.5)	80 (75.5)	**0.010**
	**Yes**	10 (10.5)	26 (24.5)	
**Temporomandibular joint syndrome *n* (%)**	**No**	93 (97.9)	95 (89.6)	**0.017**
	**Yes**	2 (2.1)	11 (10.4)	
**Migraine or tension headache *n*** *(%)*	**No**	86 (90.5)	80 (75.5)	**0.005**
	**Yes**	9 (9.5)	26 (24.5)	
**İrritable bowel syndrome** *n (%)*	**No**	84 (88.4)	74 (69.8)	**0.001**
	**Yes**	11 (11.6)	32 (30.2)	
**Chemical sensitivity** *n (%)*	**No**	92 (96.8)	101 (95.3)	0.724
	**Yes**	3 (3.2)	5 (4.7)	
**Neck injury (including Whiplash)** *n (%)*	**No**	85 (89.5)	101 (95.3)	0.118
	**Yes**	10 (10.5)	5 (4.7)	
**Anxiety** *n (%)*	**No**	80 (84.2)	73 (68.9)	**0.011**
	**Yes**	15 (15.8)	33 (31.1)	
**Depression** *n (%)*	**No**	87 (91.6)	76 (71.7)	**< 0.001**
	**Yes**	8 (8.4)	30 (28.3)	

Table 4 shows a further correlation analysis between the CSI‐A global score and clinical parameters of NCM patients. This correlation matrix revealed a statistically significant correlation between age, duration of pain, and the scores of SF‐MSQ, including sensory and affective dimensions and total breast pain score; PPI, VAS, Pain Detect; LANSS; HADS‐t; HAD‐A; HAD‐D; all the NHP‐1 subgroups; NHP‐2; and CSI‐A global score (*p* < 0.001).

**TABLE 4:** Correlation between the CSI‐A global score and clinical parameters of NCM patients.

	CSI—A (*n* = 106)	
	*r*	*p*
**Age**	−0.237	**0.014**
**BMI**	0.113	0.248
**Duration of pain**	0.585	**< 0.001**
**McGill Pain Questionnaire %**		
Sensory dimension %	0.752	**< 0.001**
Affective dimension %	0.585	**< 0.001**
Total breast pain score %	0.746	**< 0.001**
**PPI**	0.679	**< 0.001**
**VAS**	0.665	**< 0.001**
**Pain Detect**	0.853	**< 0.001**
**LANSS**	0.736	**< 0.001**
**HAD Anxiety**	0.679	**< 0.001**
**HAD Depression**	0.533	**< 0.001**
**HAD Total**	0.715	**< 0.001**
**Nottingham Health Profile Part 1**	0.788	**< 0.001**
**Pain**	0.575	**< 0.001**
**Emotional reaction**	0.528	**< 0.001**
**Sleep**	0.466	**< 0.001**
**Social isolation**	0.634	**< 0.001**
**Physical mobility**	0.539	**< 0.001**
**Energy**	0.623	**< 0.001**
**Nottingham Health Profile Part 2**	0.711	**< 0.001**

According to the results of the linear regression analysis constructed based on disease characteristics to identify the factors that predicted CSI‐A score, duration of pain, PAIN‐Detect, and the sensory dimension of McGill Questionnaire (dependent variables) with CSI‐A score (independent variable) were found to be the most significant factors, showing the positively direct proportion with the increase in CSI‐A score (*p* = 0.014, *p* < 0.001, and *p* < 0.001, respectively (Table 5)).

**TABLE 5:** Linear regression analysis of age, duration of pain, PAIN‐Detect, and sensory dimension of the McGill Questionnaire (dependent variables) with CSI‐A score (independent variable).

	*B*	SE	*t*	*p*
**Constant**	−7.863	4.255	−1.848	0.068
**Age**	−0.127	0.102	−1.245	0.216
**Duration of pain**	0,175	0.070	2.502	**0.014**
**PAIN‐Detect**	1.019	0.096	10.592	**< 0.001**
**Sensory dimension %**	0.341	0.057	6.024	**< 0.001**

*R^2^* = 0.810; ANOVA F = 107.47; p < 0.001.

## 4 Discussion

In this study, we investigated the relationship between CSS criteria and sub‐parameters with pain severity, psychosocial status, and QoL in patients with NCM. The results of this study indicate that NCM may be regarded as an integral component of CSS, with pain intensity and the psychosocial well‐being as well as the QoL being profoundly influenced in women displaying the hallmark features of these syndromes.

NCM patients had significantly higher CSI‐A scores than controls, indicating a strong relationship between NCM and CSS. Literature highlights that PS in chronic pain may progress to CS (Staud and Smitherman 2002; Graven‐Nielsen and Arendt‐Nielsen 2002). Woolf et al. (Woolf 2011) emphasized the critical role of CS processes in various chronic pain disorders, including fibromyalgia and chronic fatigue syndrome. Based on our findings, we strongly advocate the idea that NCM should be considered alongside other syndromes under the CSS framework. In other words, our study results emphasize that NCM shares similar pathophysiological mechanisms with other syndromes under the CSS‐related conditions, warranting its consideration alongside these syndromes. The literature shows that NCM shares common features with other syndromes included in CSS, such as fibromyalgia, inflammatory bowel diseases (IBD), irritable bowel syndrome (IBS), temporomandibular joint disorders (TME), and chronic fatigue syndrome, and that the likelihood of co‐occurrence is high. All of the aforementioned pain syndromes categorized under the heading of CSS are considered disorders in which these concerned complex pathophysiological mechanisms are actively involved. This mechanism interferes with a subjective pain that the patient is unable to clearly localize or describe in detail, characterized by enhanced responsiveness of nociceptive neurons in the CNS to normal or subthreshold sensory input, resulting in persistent pain amplification and altered pain perception. Numerous studies in the literature characterize NCM as a clinical manifestation that closely parallels the pathophysiological mechanisms observed in other chronic pain syndromes, with patients often describing their symptoms in a manner that reflects the sequential stages of CS. For instance, Clauw et al. indicated that NCM shares common pathophysiological mechanisms with other syndromes within CSS, such as fibromyalgia (Clauw 2014). Similarly, Falling et al. reported that central sensitization processes play a significant role in individuals with IBD. They observed a parallel increase in musculoskeletal pain, which is thought to result from similar sensitization mechanisms involved in the activation of disease symptoms (Falling et al. 2020). As noted in the literature, this patient group experiences a much higher frequency of chronic pain subtypes across the entire spectrum compared to the control group. On the other hand, in the study by Verne et al., an increase in both cutaneous and visceral pain sensitivity was observed in nearly all patients with irritable bowel syndrome, with a notable rise in musculoskeletal and soft tissue sensitivity, including chest and breast pain, occurring during periods of disease activation (Verne et al. 2001). Aaron and Buchwald examined the co‐occurrence of other CSS syndromes in individuals with TME and found that in the patient group with TMD, a significant increase in fibromyalgia and IBD was observed, just as seen in NCM (Aaron and Buchwald 2001). All these findings support the inclusion of NCM within the CSS framework, emphasizing the need for a multidisciplinary approach in its management. Dynamic treatment modalities prepared by addressing all accompanying pain syndromes together have been found effective in both alleviating symptoms and prolonging the period without relapse. A study by Häuser et al. (2013) clearly emphasizes the effectiveness of multidisciplinary approaches in managing syndromes associated with CS and highlights the importance of dynamic approaches with active participation for effective treatment. Last but not least, Basim and Tolu are the first authors in the literature to suggest that NCM may not be a standalone symptom but rather a component of a broader chronic pain syndrome, namely CSS. This hypothesis was supported by their analysis of sleep patterns and quality in affected patient populations. In line with their findings, they emphasized the need for further research into treatment approaches for NCM, which may differ significantly from currently accepted and applied methods (Basım and Tolu 2022). In this context, it is reasonable to emphasize the importance of evaluating NCM in parallel with other syndromes associated with CS while supporting it with multidisciplinary treatment strategies to ensure a rapid, effective, and long‐term treatment outcome.

Furthermore, in our study, NHP‐1, NHP‐2, and HADS total scores in NCM patients were found to be significantly higher compared to the control group. This indicates that NCM is not limited to physical pain alone but also negatively affects the QoL and psychosocial status of patients. Anxiety, depression, and sleep disorders are more commonly seen in NCM patients, and these conditions are known to increase pain sensitivity and further impact daily life (Katar and Başer 2021; Kanat et al. 2016). Similarly, a study by Watson et al. (2015) found that 60% of patients with chronic mastalgia exhibited symptoms of anxiety and depression, significantly worsening their pain perception. Psychosocial factors play a critical role in the progression from PS to CS in NCM, as seen in other CSS. This leads to a recurring pain‐anxiety‐pain cycle, resulting in a persistent pain syndrome that negatively impacts QoL and reduces the effectiveness of treatments focused solely on pain relief. The presence of this vicious cycle highlights the importance of addressing psychosocial factors in managing NCM to improve treatment outcomes and overall patient well‐being. A study by Gatchel et al. (2007) showed that QoL in chronic pain patients is significantly influenced by psychosocial factors such as anxiety and depression. Similarly, a study by Smith et al. found that anxiety and depression symptoms significantly worsen pain perception in mastalgia patients (Smith et al. 2004). Watson et al.’s study demonstrated that anxiety and depression are prevalent in patients with chronic mastalgia, further worsening pain perception (Watson et al. 2015).

PS typically helps the injured tissue by creating a hyperalgesic state in the affected area and its surroundings, forming a self‐protective mechanism until the injury heals. However, prolonged PS is also a pathological pain mechanism, and there is still an opportunity to take measures to prevent the chronicity of the sensitization state. Whereas in central sensitization, pain occurs even without peripheral input, and in fact, specifically, nociceptor stimuli can induce a prolonged yet reversible enhancement of neuronal excitability and synaptic efficacy within central nociceptive pathways. At this point, the literature has increasingly highlighted treatment models that aim to reduce anxiety levels, as well as improve QoL and sleep disturbances, rather than focusing solely on alleviating centrally sensitized pain in chronic pain syndromes. These approaches emphasize the modulation of glial activators as a central component of management (Graven‐Nielsen and Arendt‐Nielsen 2002; Woolf 2011; Gatchel et al. 2007; Nijs et al. 2017). This finding aligns with the high HADS scores observed in NCM patients in our study and suggests that psychosocial support may be an important component for these patients. Additionally, a meta‐analysis by Häuser et al. (2009) demonstrated that multidisciplinary approaches in the management of CSS are effective in improving patients’ overall QoL and managing pain. Similarly, Nijs et al. (2019) emphasized the efficacy of multidisciplinary approaches in the management of CSS, consisting of a personalized multimodal treatment plan that incorporates pain neuroscience education, cognition‐targeted exercise therapy, sleep management, stress management, and/or dietary interventions. They highlighted this approach as a crucial tool for enhancing the overall QoL of patients. Pain management, psychosocial support, and lifestyle modifications may also play important roles in the management of NCM.

The study's finding that NCM may be a component of CSS provides researchers with a novel conceptual framework that has significant implications for both diagnostic classification and therapeutic intervention. The eventual recognition of NCM as a pain syndrome that extends far beyond the underlying pain mechanism, and its potential treatability, similar to other sensitized pains, could reduce unnecessary hospital visits due to patients' unwarranted fears, as well as the repeated and unnecessary breast imaging conducted during research on this persistent pain. Given that NCM also progresses into a chronic process through sensitization mechanisms, various medical and non‐medical treatment methods, similar to those applied to other components of CCS, may find their place in clinical practice. The literature includes studies including both medical and non‐medical approaches to treat various forms of neuropathic pain that become chronic through sensitization (Kataria et al. 2024). Many of these studies identify fibromyalgia as the primary and shared component of the pain, with treatment strategies often defined through pathways rooted in the etiology of fibromyalgia (Rugnath et al. 2024; Hu et al. 2024; Hedman‐Lagerlöf et al. 2024). Some neuromodulator agents, including gabapentin and tricyclic antidepressants (TCAs), in clinical use despite challenges in optimizing their therapeutic window, are used alongside serotonin‐norepinephrine reuptake inhibitors (SNRIs) as first‐line medical options for achieving safe pain control in this patient population. Moreover, their potential application in patients with mastalgia has been widely studied (Jenkins et al. 1993; Sasaki et al. 2018). Similarly, studies suggest that atypical antipsychotics may serve as a therapeutic option for managing severe pain by disrupting the vicious cycle pathways involved in pain sensitization (Jimenez et al. 2018). On the other hand, various integrative approaches, including dry needling and platelet‐rich plasma therapy, as well as dietary and nutritional support programs, have been effectively utilized in these neuropathic pain pathways. These alternative medical approaches, which may serve as available options to conventional medical treatments, are increasingly reflected in daily practice, particularly for selected components of CSS (Sedighimehr et al. 2024; Badaeva et al. 2024). In this context, the use of evidence‐based psychological treatment modalities, particularly for centralized pain, is frequently highlighted in the literature (Ferrillo et al. 2022; Kim et al. 2006; Suzuki et al. 2021). Moreover, this method has been thoroughly evaluated, and it is frequently applied in pain management modalities (Lumley and Schubiner 2019). Considering that the brain not only modulates but also generates pain, and acknowledging the hypothesis that NCM establishes a form of centralized pain, the concept of combining such cognitive therapies with the aforementioned integrative approaches offers promising projections for the future. The increasing healthcare expenses and the frequency of repeated radiological examinations for NCM patients, as well as the wide spectrum of medications used, from those with minimal to more significant side effects, make it clear that the application of these treatment modalities will result in a noticeable improvement in patients' QoL.

The present study is not without its limitations. First, the cross‐sectional design restricts the ability to draw causal inferences between NCM and CS. Second, the relatively small sample size necessitates validation of the findings through larger and more prolonged studies to enhance the robustness of the results. Thirdly, although the questionnaires and assessment tools used in the study are validated, they remain subjective by nature and may introduce bias. Last, future investigations should focus on examining the neuroinflammatory aspects of NCM and their association with clinical outcomes through longitudinal study designs.

On the other hand, the distinction of the present study lies in its potential to challenge the established perception in the literature, where NCM has traditionally been regarded as a condition exclusively related to the breast, with the breast itself being the primary target organ in treatment. This study suggests that NCM can be considered a component of CSS, emphasizing the need for diversified treatment methods to improve patients’ QoL and psychosocial status. Developing multidisciplinary approaches and comprehensive treatment strategies in the management of NCM, along with a comprehensive treatment plan that includes screening for other CSS subgroups, is crucial for improving patients' QoL. Exploring the efficacy of regenerative treatment methods, including platelet‐rich plasma (PRP) may offer promising new avenues for managing NCM. Future studies are essential to better understand the underlying mechanisms of NCM and to develop innovative treatment strategies that improve patient outcomes.

## Author Contributions

Concept—P.B,S.T.; Design—P.B,S.B.; Supervision—P.B., S.T; Resources –P.B., C.K.P, P.A; Data Collection and/or Processing –P.B., P.A.; Analysis and/ or Interpretation –S.T.; Literature Search—P.B., C.K.P, P.A;; Writing Manuscript—P.B., P.A; S.B.; Critical Review—P.B.; S.B; S.T

## Ethics Statement

Ethical approval for the study was received from the Research Ethics Committee of Medipol University (December 30, 2020, with the reference number E‐10840098‐772.02‐66560).

## Conflicts of Interest

The authors declare no conflicts of interest.

## Peer Review

The peer review history for this article is available at https://publons.com/publon/10.1002/brb3.70962.

## Data Availability

The data that supprt the findings of this study are available from the corresponding author (P. Basim) upon request. This manuscript was subject to Transparent Peer Review, and the peer review reports are available at the journal's website.
